#  Molecular and cellular characterization of ABCG2 in the prostate

**DOI:** 10.1186/1471-2490-7-6

**Published:** 2007-04-10

**Authors:** Laura E Pascal, Asa J Oudes, Timothy W Petersen, Young Ah Goo, Laura S Walashek, Lawrence D True, Alvin Y Liu

**Affiliations:** 1 Department of Urology, and the Institute for Stem Cell and Regenerative Medicine, University of Washington, Seattle WA 98195, USA; 2 Institute for Systems Biology, Seattle WA 98103, USA; 3 Department of Pathology, University of Washington, Seattle, WA 98195, USA

## Abstract

**Background:**

Identification and characterization of the prostate stem cell is important for understanding normal prostate development and carcinogenesis. The flow cytometry-based side population (SP) technique has been developed to isolate putative adult stem cells in several human tissue types including the prostate. This phenotype is mainly mediated by the ATP-binding cassette membrane transporter ABCG2.

**Methods:**

Immunolocalization of ABCG2 was performed on normal prostate tissue obtained from radical prostatectomies. Normal human prostate SP cells and ABCG2^+ ^cells were isolated and gene expression was determined with DNA array analysis and RT-PCR. Endothelial cells were removed by pre-sorting with CD31.

**Results:**

ABCG2 positive cells were localized to the prostate basal epithelium and endothelium. ABCG2^+ ^cells in the basal epithelium constituted less than 1% of the total basal cell population. SP cells constituted 0.5–3% of the total epithelial fraction. The SP transcriptome was essentially the same as ABCG2^+ ^and both populations expressed genes indicative of a stem cell phenotype, however, the cells also expressed many genes in common with endothelial cells.

**Conclusion:**

These results provide gene expression profiles for the prostate SP and ABCG2^+ ^cells that will be critical for studying normal development and carcinogenesis, in particular as related to the cancer stem cell concept.

## Background

Experimental evidence suggests that prostatic epithelial stem cells exist and are likely localized to the basal epithelium [1]. Basal, luminal secretory and a small population of neuroendocrine cells constitute the epithelial component of prostatic acini. Basal and luminal cells may belong to two functional cell types descended from a common stem cell type. We are interested in identifying and isolating this prostatic stem cell. Studies to date suggest that stem cells from diverse tissue sources may contain a common set of gene transcripts, which are required for maintenance of the stem cell phenotype [2]. Considerable research efforts have been directed towards discovery of markers associated with the putative prostate stem cell, including the side population (SP) phenotype [3], integrin α_2_β_1 _(CD49b/CD29) [4,5] and PROM1 (CD133) [6]. Identification and characterization of a stem/progenitor cell population is important to our understanding of not only normal prostate development but also the cancer process, particularly in regard to cancer stem cells [7]. This knowledge may lead to the development of effective cancer treatment strategies such as differentiation and cell-based therapy.

The ATP-binding cassette membrane transporter ABCG2 (BCRP/Bcrp1) functions as an energy-dependent efflux pump, and was first identified in the breast cancer cell line MCF-7 [8]. ABCG2 is highly expressed in human endothelial cells and plays an important role in the blood-brain barrier [9-11], but it is rarely expressed in most other differentiated cell types [12]. Expression of ABCG2 is associated with multi-drug resistance; more significantly, ABCG2 is the molecular determinant for the SP phenotype and has been postulated as a universal stem cell marker [13]. Goodell et al. discovered a small and distinct SP of whole bone marrow cells based on their capacity to efflux the fluorescent dye Hoechst 33342 [14]. Remarkably, although this SP comprised ~0.1% of total bone marrow cells, it accounted for virtually all of the hematopoietic stem cell (HSC) activity as demonstrated by bone marrow repopulation assays [15]. Subsequent studies of ABCG2-null mice have attributed this dye efflux to expression of ABCG2 [13]. Since the initial discovery of the hematopoietic SP, an analogous population has been detected in embryonic stem cells, the liver, heart, and many other organs including the prostate [3,13,16,17]. Collectively, these studies provide evidence that the SP phenotype, and therefore ABCG2 expression, may represent a feature shared by stem cells of different tissue origins. However, other recent studies have found no direct correlation between SP cells and ABCG2 expression [18]. Both SP and known stem/progenitor cells express other ABC transporters including ABCB1 (MDR-1), ABCC1 and ABCA2, suggesting that these latter molecules may also be involved in determining the SP phenotype [19-21].

ABCG2 expression in the prostate has been reported in both the epithelium [22] and endothelium [23]. The SP of the prostate has been previously isolated and characterized as integrin α2^+ ^and containing a subpopulation of quiescent (~12%) cells [3]. Immunohistochemical analysis of both normal and cancerous ABCG2^+ ^cells shows that this subset also lacks the androgen receptor (AR) protein, and it has been proposed that ABCG2-mediated efflux of androgen is a mechanism for maintenance of the prostate stem cell phenotype [24].

In the cancer stem cell model, tumors are thought to contain phenotypically diverse populations of cancer cells, but only a minority of these cells (10–35%) possess the ability to form new tumors [7]. It is postulated that these cancer "stem" cells drive tumor growth and expansion, and are resistant to therapy. For breast cancer tumors, it was found that as few as 100 tumorigenic (CD44^+^/CD24^-/low^) cells could form new tumors that contained both the tumorigenic and non-tumorigenic cell types [25]. These cancer cells, like stem cells, can self-renew as well as "differentiate" into other cancer cell types to produce tumor heterogeneity.

The goal of this study was to determine if the SP of normal human prostate and ABCG2^+ ^cells are similar populations that express putative stem cell markers. Furthermore, we sought to more precisely define the patterns of ABCG2 expression in prostate tissue, determine if the non-endothelial ABCG2 expressing cells could be isolated, and finally to characterize those cells by comparing the transcriptome from sorted SP cells with that of cells isolated using an antibody (5D3) that recognizes an external epitope of human ABCG2 [26]. This transcriptome data can be used for future comparison with candidate cancer stem cells isolated from prostate tumors.

## Methods

### Cell lines and prostate tissue

The breast cancer cell line MCF-7 and prostate cancer line C4-2 were obtained from American Type Culture Collection (Manassas, VA) and cultured in RPMI-1640 (Cambrex BioScience, Walkersville, MD) media supplemented with 10% heat-inactivated fetal bovine serum (FBS). The tissue samples that were used in this study consisted of cancer-free samples obtained from 30 radical prostatectomies. All tissue samples were obtained under approval by the University of Washington Institutional Review Board. Samples of cancer-free prostate parenchyma were collected following a standard protocol. To minimize RNA degradation, upon receipt of a radical specimen, 3-mm thick transverse sections were made of the prostate after inking the exterior surface (the surgical margin). Between 2 and 10 g of tissue from the anterior aspect of the prostate (transition zone) were excised. A frozen section of a block of tissue corresponding to that non-frozen tissue from which the cells were excised was taken and histologically assessed to confirm that the specimen was free of cancer. The sample was minced and digested by overnight incubation at room temperature in an aqueous solution of 0.2% collagenase type I (Invitrogen, Carlsbad, CA) in RPMI-1640 media supplemented with 5% FBS and 10^-8 ^M dihydrotestosterone on a magnetic stirrer. The resultant cell suspension was filtered with a 70-μm Falcon cell strainer to remove non-digested tissue, diluted with an equal volume of Hanks balanced salt solution (HBSS), and aspirated with an 18-gauge needle. The resultant predominantly single cell preparation was partitioned into stromal and epithelial fractions on a discontinuous Percoll density gradient (Amersham Pharmacia, Piscataway, NJ) as described previously [27,28]. High purity is obtained by this method [27]. Cells banding at a density of ρ = 1.07 were collected as the epithelial cell fraction and used for cell sorting. Because stromal cells (ρ = 1.035) are less dense they cannot sediment into the epithelial density band and the epithelial fraction is therefore relatively free of stromal cells.

### Immunohistochemistry

Blocks of unfixed prostate tissue, typically from the peripheral zone where cancer arises, were harvested at the time of surgery. Serial 5-μm sections were prepared from randomly selected frozen blocks, fixed in cold acetone, and processed for immunohistochemistry. Immunohistochemistry was performed as described previously, using a three-step indirect avidin-biotin-peroxidase procedure [29]. The primary antibodies used were mouse monoclonal anti-ABCG2 (BXP-34, Chemicon, Temecula, CA) diluted 1:100 in phosphate-buffered saline (PBS), and CD138 (MI15, BD-PharMingen, San Diego, CA) diluted 1:100 in PBS. Antigen was localized using biotinylated anti-mouse IgG (BA-2000, Vector Labs, Burlingame, CA) as the secondary antibody, and diaminobenzidine tetrahydrochloride was the chromogen. The sections were counterstained in hematoxylin. Immunostained sections were imaged with an Olympus BX41 microscope (Olympus, Melville, NY) equipped with a MircoFire digital camera (Optronics, Goleta, CA). Percentage of ABCG2^+^/CD138^+ ^basal cells was obtained by manual counting of 5 representative CD138^+ ^glands (approximately 1 in 20 glands in a field contained ABCG2-positive basal epithelial cells). Composite images were constructed with Photoshop CS (Adobe Systems, San Jose, CA).

### Hoechst 33342 labeling and SP sorting

The epithelial cell fraction enriched by Percoll gradient was resuspended at a concentration of 10^6 ^cells/ml in HBSS, supplemented with 10% FBS, 20 mM N-2-hydroxyethylpiperazine-N'-2-ethanesulfonic acid (HEPES), pH 7.4, and 1% D-glucose. Hoechst 33342 (Molecular Probes, Eugene, OR) was added to a concentration of 2–5 μg/ml. Cells were incubated at 37°C for 90 min and then placed on ice until analysis. Fluorescence-activated cell sorting (FACS) was done using a high-speed cell sorter (InFlux, Cytopeia, Seattle, WA) following the protocol of Goodell et al. [14]. Fluorescence from the Hoechst-stained cells was excited by 100–150 mW UV from an Innova 305c argon ion laser (Coherent, Santa Clara, CA). Fluorescence was monitored at two emission bands from 400 – 480 nm (Hoechst blue) and from 690 – 800 nm (Hoechst red). Epithelial cells were sorted by magnetic cell sorting (MACS, Miltenyi Biotech, Auburn, CA) for CD44^+ ^(basal) cells prior to SP sort. In MACS sorting, cells were resuspended in 0.1% bovine serum albumin (BSA)-HBSS and labeled with phycoerythrin (PE)-conjugated CD44 antibody (BD-PharMingen). After the primary antibody, the cells were incubated with anti-PE microbeads (Miltenyi Biotec). The cell suspension was filtered and then sorted by using the "DOUBLE POSITIVE SORT" program of an AutoMACS system (Miltenyi Biotec). Both the positive and negative fractions were analyzed by FACS to assess sorting efficiency using a FACSCalibur flow cytometer (Becton Dickenson, Mountainview, CA). The selected epithelial cells were then sorted for SP. The sorted SP cells were lysed in RLT Buffer (Qiagen, Germantown, MD) and RNA was isolated using RNeasy kits (Qiagen). The RNA quality was monitored by using Agilent 2100 Bioanalyzer with RNA Nano Labchip (Agilent Technologies, Palo Alto, CA). Some degradation was evident, likely due to the SP sorting process.

### MACS isolation of ABCG2^+ ^cells

The epithelial cell fraction was resuspended in 0.1% BSA-HBSS, and labeled with anti-CD31-PE followed by anti-PE microbeads. This step was intended to first remove ABCG2^+^/CD31^+ ^endothelial cells. The CD31-negative fraction was then incubated with anti-ABCG2-PE. The reaction was stopped by the addition of 1 ml 0.1% BSA-HBSS and centrifugation. The labeled cells were resuspended and 15 μl paramagnetic microbead-conjugated anti-PE antibody was added for 15 min. Afterwards, the positive and negative cells were separated with an AutoMACS using the "DOUBLE POSITIVE SORT" program. Aliquots of the positive and negative cell fractions were analyzed by FACS to assess sort efficiency. The ABCG2-positive cells were pelleted by centrifugation and resuspended in RLT for storage at -80°C. RNA was isolated and the RNA quality was monitored. Only RNA samples that were of sufficient concentration and showed no degradation were used for array hybridization.

### Affymetrix expression profiling

Five separate biological replicates of the sorted cell population were assayed to produce a dataset using the Human Genome U133 Plus 2.0 GeneChips (Affymetrix, Santa Clara, CA). The U-133 Plus 2.0 array contains probesets representing 54,675 genes, splice variants, and ESTs. In addition to the SP and ABCG2^+ ^cells, transcriptomes of the following prostate cell types have been previously determined: CD104^+ ^(basal epithelial) cells, CD26^+ ^(luminal epithelial) cells, CD49a^+ ^(stromal) cells, and CD31^+ ^(endothelial) cells [30]. The GeneChips were prepared, hybridized, and scanned according to the protocols provided by Affymetrix. Briefly, 200 ng of RNA was reverse transcribed with poly (dT) primer containing a T7 promoter, and the cDNA was made double-stranded. In vitro transcription was performed to produce unlabeled cRNA. Next, first-strand cDNA was produced with random primer. cDNA was made double-stranded with poly (dT) primer/T7 promoter. Finally, in vitro transcription was performed with biotinylated ribonucleotides. The biotin-labeled cRNA was hybridized to the GeneChips. The chips were washed and stained with streptavidin-PE using an Affymetrix FS-450 fluidics station. Data was collected with an Affymetrix GeneChip Scanner 3000.

### Data analysis

Comparative analysis between the SP, ABCG2^+ ^and the other cell-type datasets was used to identify genes specific to stem cells. CEL files produced by GeneChip Operating Software (GCOS, Affymetrix) were loaded into GeneSpring 7.2 (Agilent) via the robust multiple array average (RMA) preprocessor. The GeneSpring RMA preprocessor uses the same analysis algorithm for Affymetrix array data as the open source Bioconductor project. GeneSpring's replicate error model was used for our analysis. Genes in the dataset with an average raw fluorescence signal <50 were considered to be undetected by the experiment. Statistical significance of differential expression between the various cell sorts was determined by 1-way ANOVA at p < 0.05.

### Gene expression validations

Reverse transcriptase-polymerase chain reaction (RT-PCR) was used to validate expression. For cell lines and tissue specimens, 1 μg RNA was reverse transcribed with superscript II reverse transcriptase (Invitrogen, Carlsbad, CA) at 50 C for 50 mins followed by 10 min at 70 C. Gene-specific primers for PCR (Table 1) were designed to produce amplicons from 100–650 bp in size. PCR was carried out at 95 C 30 s, 55 C 30 s, 72 C 1 min for 35 cycles. PCR products were resolved on 2% agarose gels. The ribosomal gene RPL13a served as the internal reference for each sample. Quantitative real-time PCR was performed by using SYBR Green I on ABI 7900HT (Applied Biosystems, Foster City, CA). Up to 1 μg of RNA from CD31^+ ^and ABCG2^+ ^(5D3^+^) cells were used for cDNA synthesis. The cDNA was then used as template for real-time PCR with gene specific primers. The quantitative PCR (qPCR) assay was run in a 96-well Optical Reaction Plate (Applied Biosystems) with 20 μl final volume per well. Each sample was run in triplicate for each gene. The reaction contained 3–5 μl template cDNA, 10 μl SYBR Green PCR Master Mix (Applied Biosystems) and 10 pmol each of forward and reverse primer. Gene-specific primers for qPCR were designed using Primer Express™ 1.5 (Applied Biosystems) (Table 2). Primer Sets were initially validated for equal amplification efficiencies of the genes. qPCR was carried out at 95 C 30 s, 55 C 30 s, 72 C 1 min for 40 cycles with RPL13a as the internal reference for each sample. The comparative Ct method was used to assess relative mRNA levels between two samples using CD31^+ ^cells as a calibrator basis for comparison.

## Results

### Immunolocalization of ABCG2^+ ^epithelial cells to the basal epithelium

**Figure 1:**
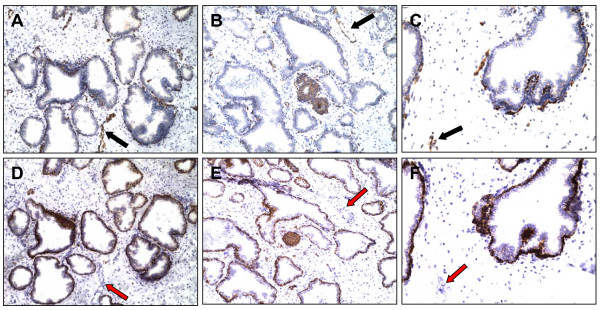
**ABCG2^+ ^cells in the prostate epithelium**. Serial sections of normal human prostate were stained for ABCG2 **(A – C) **or CD138 **(D – F)**. A subpopulation of cells in the basal epithelium is ABCG2^+^. Endothelial cells of capillaries (black arrow) are also ABCG2^+ ^but are CD138^- ^(red arrow). All basal cells of a particular small gland were ABCG2^+^/CD138^+ ^**(B)**. Original magnification is 100×, magnification for **C **and **F **is 200×.

Fig. 1 shows the result of prostate immunostaining of ABCG2 and the prostate basal epithelial marker CD138 (syndecan 1) [29]. The ABCG2 antibody recognizes an internal epitope of the human ABCG2 protein [31]. All basal cells of a particular acinus were labeled by CD138 but only about 3% of these cells were also positive for ABCG2. Most glands had very few or no ABCG2^+ ^cells. Furthermore, distribution of the ABCG2-positive cells within the basal epithelium consisted of both single cells and cell clusters. Three cell types could be distinguished: ABCG2^+^/CD138^+ ^basal cells, ABCG2^-^/CD138^+ ^basal cells, and ABCG2^+^/CD138^- ^endothelial cells of vessels, which are known to express ABCG2 [11].

### Prostate epithelial SP

**Figure 2:**
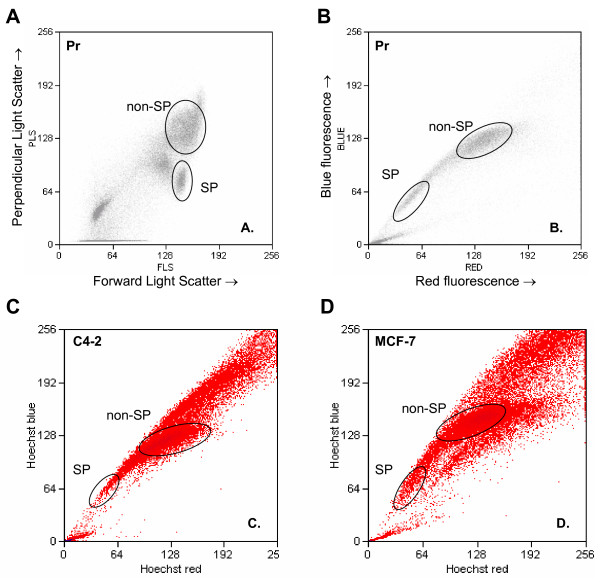
**Prostate epithelial side population**. Prostate cells prepared from tissue specimens were analyzed for Hoechst 33342 dye efflux. The cytograms show **(A) **forward and side light scatter of the SP and non-SP populations as defined by **(B) **the dye efflux characteristics of the cells. Cytograms show the dye efflux characteristics of **(C) **the prostate cancer cell line C4-2 and **(D) **the breast cancer cell line MCF-7 used as positive controls.

Hoechst 33342 labeling and analysis of human prostate epithelial cells by FACS detected a distinct SP as shown in Fig. 2. The prostate SP constituted 0.5–3% of the total epithelial cell fraction. Light scattering analysis of the SP cells (Fig. 2A) indicated that they were smaller and less granular than the non-SP cells, which is characteristic of stem cells [32,33]. The Hoechst profile was plotted with BLUE on the y-axis and RED on the x-axis (Fig. 2B). The SP sorting protocol was also used on two ABCG2^+ ^cancer cell lines (MCF-7 and C4-2) as positive controls (Fig. 2C and 2D respectively).

### Prostate epithelial ABCG2^+^

**Figure 3:**
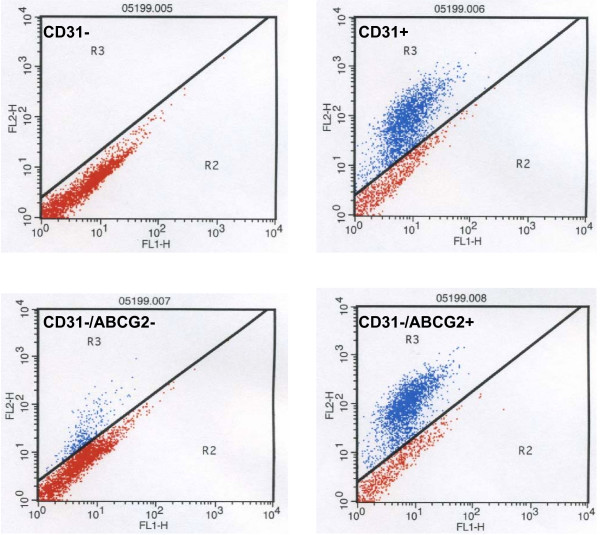
**Flow analysis of prostate epithelial cells**. Epithelial cells collected on a Percoll density gradient were stained with CD31 and ABCG2. Cells in R3 of each cytogram were sorted for further analysis.

CD31 labeling and sorting of prostate epithelial cells by MACS resulted in removal of 99.94% CD31-positive cells (Fig 3). The CD31 negative fraction was subsequently sorted using ABCG2 (Fig. 3).

### Prostate SP, ABCG2^+^, and endothelial cell transcriptomes

**Figure 4:**
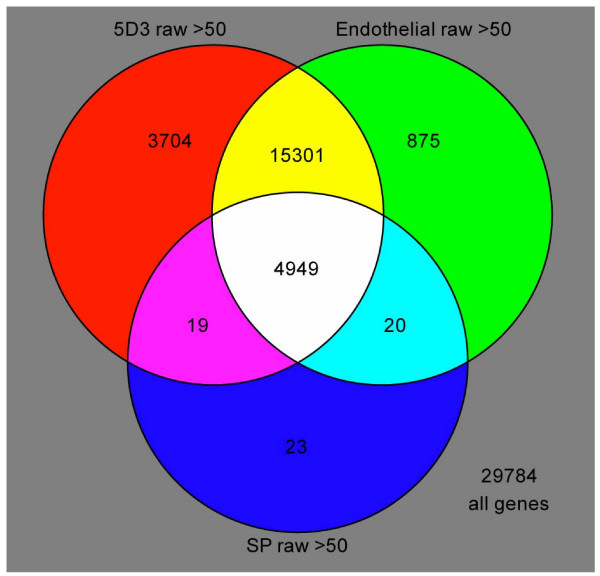
**Overlap of prostate cell transcriptomes**. The Venn diagram depicts shared genes expressed by each cell type: ABCG2^+ ^(5D3) are red, endothelial (CD31^+^) are green, and SP are blue. Other colors show genes that are shared between the cell types, and white shows genes shared by all three.

Analysis of the transcriptomes of prostate SP and ABCG2^+ ^cells revealed that the SP cell population was not significantly different from the ABCG2^+ ^population. Fig. 4 shows the expression overlap of SP, ABCG2^+^, and endothelial cell transcriptomes in a Venn diagram. The SP transcriptome contained 5,011 probesets while the ABCG2^+ ^transcriptome contained 23,973 probesets, and the endothelial transcriptome contained 21,145 probesets. Of the probesets detected in the SP transcriptome 23 were not detected in the ABCG2^+ ^or endothelial datasets. However, the low number of probesets present in the SP data suggests that RNA quality (perhaps due to Hoechst toxicity) may have significantly affected the number of genes detected. Overall, the ABCG2^+ ^transcriptome contained 3,704 probesets not detected in the SP or endothelial data. In addition to ABCG2, all three populations contained other ABC transporters including ABCE1, ABCB1, ABCG1 and ABCE1. The expression of stem cell markers detected by the arrays for each population is listed in Table 3. Of these, NR6A1 and NES were detected in the SP, ABCG2^+ ^and CD31^+ ^cell populations; and BMP-4, POU5F1, KIT and VM were detected in the ABCG2^+ ^and CD31^+ ^populations. Differentiation associated genes keratin 18 (K18), prostate acid phosphatase (PAP), and prostate stem cell antigen (PSCA) were downregulated in both SP and ABCG2^+ ^cell populations compared to the prostate basal epithelial (CD104^+^) and luminal epithelial (CD26^+^) cell transcriptomes.

### Molecular signature of stem cells in prostate SP cells

**Figure 5:**
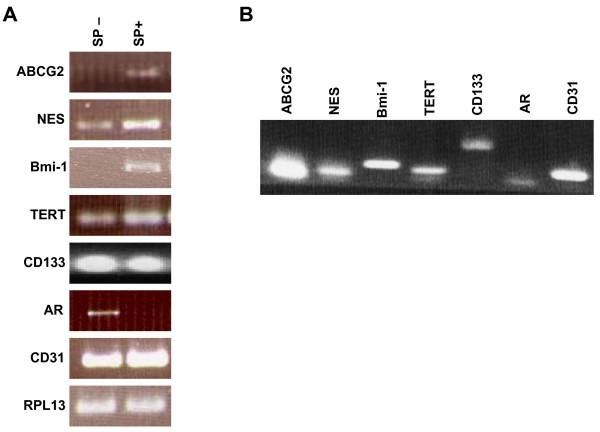
**Gene expression analysis of prostate SP cells**. **(A): **Expression of various stem cell genes in human prostate SP (SP+) vs. non-SP (SP-), by reverse transcription polymerase chain reaction (RT-PCR). The ribosomal protein-encoding gene RPL13a served as the reaction control. Nestin, telomerase, Bmi-1 are reported in the literature to be stem cell genes. CD133 has been reported as a prostate stem cell gene. Androgen receptor (AR) is known to be expressed by differentiated cells. The SP+ and SP-cells were also CD31^+^, indicating possible endothelial contamination. **(B): **Prostate endothelial cells (CD31^+^) also expressed these genes.

**Figure 6:**
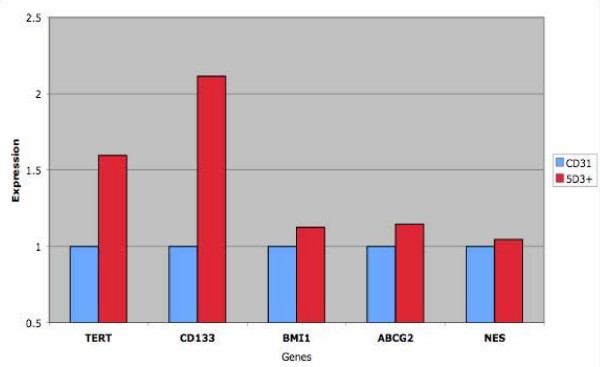
**Expression of stem cell genes in ABCG2^+ ^cells**. Expression level is relative to CD31^+ ^cells. Enrichment of TERT, CD133, BMI-1, NES and ABCG2 in the ABCG2^+ ^cells vs. CD31^+ ^was verified.

SP and non-SP cells were analyzed by RT-PCR. Analysis of the sorted SP cells showed that they were enriched in ABCG2 expression in comparison to the non-SP, and were AR^- ^(Fig. 5A). In addition to ABCG2, the SP cells were shown to differentially express the following genes: (a) nestin (NES), a marker first identified in neural stem cells [34], and subsequently in other stem cell types, with expression higher in SP than non-SP; (b) telomerase (TERT), a known marker of stem cells, again higher in SP than non-SP; and (c) BMI-1, a transcription repressor specific for the induction of telomerase in epithelial cells and the extension of proliferative lifespan, and for the maintenance of hematopoietic stem cells [35], with expression confined mainly to SP. RT-PCR analysis of CD31^+ ^endothelial cells also showed expression of these genes (Fig. 5B). The sorted SP cells likely still contained some CD31^+ ^(ABCG2^+^) endothelial cells as indicated by RT-PCR (Fig. 5A). Additionally, qPCR was performed on CD31^+ ^and ABCG2^+ ^cells. Increased levels of expression for TERT, CD133, BMI-1, NES and ABCG2 were evident in the ABCG2^+ ^cells vs CD31^+ ^was verified (Fig. 6). These results suggest that the SP, ABCG2^+ ^and CD31^+ ^cells each have some intrinsic expression of genes characteristic of stem cells, but that the ABCG2^+ ^sorting results in a greater enrichment of these genes than in CD31^+ ^cells.

## Discussion

Immunohistochemical analysis of ABCG2 expression identified two distinct positive populations in the prostate: an endothelial population and a nonendothelial basal epithelial population. Identification of nonendothelial expression was accomplished by comparing serial sections stained with the basal cell marker CD138 to ABCG2 staining patterns. Nonendothelial ABCG2 cells were identified as CD138^+^/ABCG2^+^. Nonendothelial staining for ABCG2 was observed in small clusters of cells in the basal epithelium of a few prostate glands. Endothelial CD138^-^/ABCG2^+ ^staining was observed in capillaries in the stroma, or at periglandular location.

Using a protocol established for mouse bone marrow SP analysis, we isolated an SP in normal human prostate tissue obtained from radical prostatectomies. The forward and side scatter plot revealed a smaller and less granular population of cells corresponding to the SP defined by the Hoechst RED/BLUE profile. Transcriptome analysis of the prostate SP was compared to previously sorted prostate cell transcriptomes [30]. In contrast to the CD antibody-sorted cell types, far fewer genes were detected in SP sorted cells. RNA integrity is critical for determining gene expression, and suboptimal RNA quality in the SP sorted cells was the probable cause of this decrease in genes detected. Hoechst 33342 is a DNA intercalating agent that results in significant cellular toxicity [36], and may have caused RNA degradation in cells isolated in this fashion. Several studies have addressed the potential toxicity of Hoechst 33342 [36,37], however, very little research has looked at the effects of the SP technique on the quality of RNA isolated from these cells. To mitigate the effects of Hoechst toxicity on RNA degradation, the monoclonal antibody, clone 5D3, was used to generate a transcriptome for the ABCG2^+ ^prostate epithelial cells. MACS was chosen as the separation method because the procedure is much faster than FACS (minutes *vs*. hours).

Transcriptome analysis of the SP and ABCG2^+ ^cells from the prostate included the endothelial (CD31^+^) population [30] since these cells also express ABCG2. As expected, all three populations (prostate SP, ABCG2^+ ^and CD31^+^) expressed higher levels of ABCG2 than the other prostate cell types: luminal, stromal, basal, and epithelial (data not shown). Additionally, all three populations contained other ABC transporters including ABCE1, ABCB1, ABCG1 and ABCE1 [see Additional file 1]. The ABCG2^+ ^(5D3) transcriptome dataset was found to contain many genes present in endothelial cells, although it did contain 232 unique probesets with a raw fluorescence signal >100 [see Additional file 2]. The sorting scheme used to isolate ABCG2^+ ^cells by presorting with CD31-PE might have been inefficient in removing all CD31^+ ^cells. However, MACS sorting of ABCG2^+ ^cells did result in a more complete transcriptome than the SP sorting method [see Additional file 3], as evidenced by the nearly 5-fold increase in number of genes identified. RT-PCR analysis revealed that SP cells expressed higher levels of the putative stem cell markers NES, TERT, and BMI-1, in addition to ABCG2 compared to non-SP cells. In the same analysis, other markers, such as the endothelial marker CD31 [29] and the prostate stem cell marker CD133 [6] were expressed at similar levels by both SP and non-SP cells. CD31^+ ^cells also expressed NES, BMI-1, TERT and CD133. qPCR analysis of ABCG2+ cells verified that the ABCG2^+ ^sorting resulted in enrichment for these genes as compared to CD31^+ ^cells. Based on these transcriptome and RT-PCR analyses, the distinction between the prostate endothelial cell and the putative prostate stem cell isolated based on SP expression is not entirely clear. The ABCG2^+ ^population shares many endothelial genes as well, however, based on transcriptome and qPCR analyses, it seems to be a more distinct population than the SP cells. Because of their expression of ABCG2, endothelial cells are a major potential contaminant of stem cells isolated by methods targeting this marker. Alternative sorting schemes to isolate purer ABCG2^+^/CD31^- ^populations are currently being explored in our lab.

Our study was not designed to provide a functional assay for these putative stem cells. The sorting methods utilized resulted in very small numbers of cells that were insufficient for *in vitro *studies. Additionally, the fact that these cells express ABCG2, which is hypothesized to efficiently pump out differentiation inducing molecules [24], may render them non-responsive to differentiation signaling. This transcriptome data has, however, provided a wealth of targets for further studies aimed at identifying the mechanisms that govern the process of self-renewal and development of prostate stem cells.

## Conclusion

Our data show that ABCG2 efflux of Hoechst 33342 or antibodies to ABCG2 could be used in isolating a subset of prostate cells that may include the prostate stem cells. The prostate cells isolated by SP and antibodies for ABCG2 have gene expression characteristics consistent with the endothelial cell phenotype and a number of stem cell markers. Because of potential RNA degradation caused by the SP process, it may be that antibody sorting based on ABCG2 expression is a better method of isolation for the putative prostate stem cell. Future studies are needed to determine more conclusively whether SP or ABCG2^+ ^results in organ-specific stem cell enrichment in the prostate, or whether other putative markers such as integrin α_2_β_1 _(CD49b/CD29) [4,5] and PROM1 (CD133) [6] are better suited for prostate stem cell isolation.

## Competing interests

The author(s) declare that they have no competing interests.

## Authors' contributions

LEP: study design, experiments, and drafting of the manuscript. AJO: gene expression array and data analysis. TWP: side population sorting. YAG: qPCR and data analysis. LSW: flow cytometry. LDT: tissue acquisition, immunohistochemistry and pathology. AYL: study conception, study design and coordination. All authors have read and approved the final manuscript.

## Pre-publication history

The pre-publication history for this paper can be accessed here:



## Supplementary Material

Additional file 1 ABC transporters in SP, ABCG2^+ ^and endothelial cell transcriptomes. Transcriptome analysis of prostate SP, ABCG2^+ ^and endothelial cells detected 19 probesets for ABC genes with a raw fluorescence signal > 100. Click here for file

Additional file 2 ABCG2 unique genes. Transcriptome analysis of prostate ABCG2^+ ^detected 1291 probesets not detected in the SP or endothelial data with a raw fluorescence signal > 50, 232 probesets with signal > 100. Click here for file

Additional file 3 SP unique genes. Transcriptome analysis of prostate SP detected 17 probesets not detected in the ABCG2^+ ^or endothelial data with a raw fluorescence signal > 50, 8 probesets with signal > 100. Click here for file

## Figures and Tables

**Table 1:** Primer sequences used for RT-PCR.

**Gene**	**Sense Primer**	**Antisense Primer**	**PCR Product**
ABCG2 [GenBank:NM_004827]	AAGCCATTGGTGTTTCCTTG	CCTGAGATCCTGAGCCTTTG	124
AR [GenBank:NM_000044]	GCAGAGTGCCCTATCCCAGTC	GGCAGTCTCCAAACGCATGT	101
BMI-1 [GenBank:NM_005180]	AAGTCTTGCCTGCTTTCCAA	CAGCGGTAACCACCAATCTT	316
PECAM1 (CD31) [GenBank:BC051822]	TGGGCCACAATCGCCTTGTCCT	TCCACATCAGCCCCACCGGAAT	130
PROM1 (CD133) [GenBank:NM_006017]	GCGTCAAGATACTTCAACGCACAGGG	CAGCTATCAATGTTGTGATGGGCTTGTC	650
NES [GenBank:BC108285]	CCTTCTCCAAGGAAACAGGGTCA	ATTTGAGGACCTGGGGACTGAGGCAC	428
RPL13a [GenBank:BC014167]	CCGAGAAGAACGTGGAGAAG	CTTTCAAGCAACTTCCGGAG	240
TERT [GenBank:NM_003219]	TTTGGCCGAGGCCTGCATGT	TGGTGGGGGTGGAAGGCAAA	252

**Table 2:** Primer sequences used for qPCR.

**Gene**	**Sense Primer**	**Antisense Primer**
ABCG2 [GenBank:NM_004827]	CACAAGGAAACACCAATGGCT	ACAGCTCCTTCAGTAAATGCCTTC
BMI-1 [GenBank:NM_005180]	AAATGCATCGAACGAGAATC	AATGAACCCTCCACAAAGCA
PECAM1 (CD31) [GenBank:BC051822]	GAGCACAGTGGCAACTACACG	GACCACGATGCTGCTGAC
PROM1 (CD133) [GenBank:NM_006017]	CATGTTTGGAGGATCTTGCTAGC	TTCCCGCACAGCCCC
NES [GenBank:BC108285]	CATTGTCCCCGTCGGTCTC	GAGGATGGACAGACGCGGC
RPL13a [GenBank:BC014167]	CCGAGAAGAACGTGGAGAAG	CTTTCAAGCAACTTCCGGAG
TERT [GenBank:NM_003219]	ACCGTCTGCAGGAGATC	GACCTGAGCTCGACGAC

**Table 3:** List of stem cell markers. Columns list the gene as detected (+) or undetected (-) in the SP, ABCG2 and CD31 cell transcriptomes.

**Gene**	**Description**	**SP**	**ABCG2**	**CD31**
ALPP	Alkaline phosphatase	-	-	-
AFP	Alpha-fetoprotein	-	-	-
BMP-4	Bone morphogenic protein	-	+	+
T	Brachyury	-	-	-
TNFRSF8	CD30	-	-	-
TDGF-1	Cripto	-	-	-
GATA-4	GATA-4	-	-	-
FOXD3	Genesis	-	-	-
NR6A1	Germ cell nuclear factor	+	+	+
HNF-4A	Hepatocyte nuclear factor-4	-	-	-
NES	Nestin	+	+	+
N-CAM	Neuronal cell-adhesion molecule	-	-	-
POU5F1	Oct-4	-	+	+
PAX6	Homo sapiens paired box gene 6	-	-	-
KIT	Stem cell factor (SCF or c-Kit ligand)	-	+	+
TERT	Telomerase	-	-	-
VM	Vimentin	-	+	+

* [38]
