# Supplementary material for: Molecular and cellular characterization of ABCG2 in the prostate
Source: BMC Urol. 2007 Apr 10;7:6. doi: 10.1186/1471-2490-7-6 (PMC1853103; doi:10.1186/1471-2490-7-6)
Supplement: Additional file 1 — ABC transporters in SP, ABCG2+ and endothelial cell transcriptomes. Transcriptome analysis of prostate SP, ABCG2+ and endothelial cells detected 19 probesets for ABC genes with a raw fluorescence signal > 100. [file 1471-2490-7-6-S1.pdf]

Probeset	5D3_raw	5D3_std	Endo_raw	Endo_std	SP_raw	SP_std	Common	Genbank
201873_s_at	743	267	420	147	30	84	ABCE1	NM_002940
209993_at	800	438	391	144	31	83	ABCB1	AF016535
208161_s_at	126	96	127	81	8	13	ABCC3	NM_020037
209247_s_at	236	116	250	125	22	32	ABCF2	BC001661
203192_at	103	33	68	22	15	13	ABCB6	NM_005689
209380_s_at	192	71	162	47	20	27	ABCC5	AF146074
204567_s_at	1241	256	331	257	105	98	ABCG1	NM_004915
218991_at	134	45	85	30	13	17	ABC1	NM_022070
202850_at	509	184	297	134	22	70	ABCD3	NM_002858
202394_s_at	136	51	108	32	13	19	ABCF3	NM_018358
209246_at	164	63	135	45	9	24	ABCF2	AF261091
207622_s_at	183	111	157	67	7	25	ABCF2	NM_005692
203196_at	291	112	251	74	22	43	ABCC4	AI948503
207623_at	156	44	56	21	6	12	ABCF2	NM_005692
211113_s_at	183	64	62	31	8	15	ABCG1	U34919
213353_at	159	53	114	35	7	20	ABCA5	BF693921
209735_at	3053	1000	1040	560	73	244	ABCG2	AF098951
201872_s_at	1027	252	480	168	48	96	ABCE1	AI002002
223320_s_at	194	63	110	55	6	24	ABCB10	AF277184

ABC genes >100 ANOVA

5D3 vs SP 04-04-06
