# Supplementary material for: Molecular and cellular characterization of ABCG2 in the prostate
Source: BMC Urol. 2007 Apr 10;7:6. doi: 10.1186/1471-2490-7-6 (PMC1853103; doi:10.1186/1471-2490-7-6)
Supplement: Additional file 3 — SP unique genes. Transcriptome analysis of prostate SP detected 17 probesets not detected in the ABCG2+ or endothelial data with a raw fluorescence signal > 50, 8 probesets with signal > 100. [file 1471-2490-7-6-S3.pdf]

Probeset	5D3_raw	5D3_std	Endo_raw	Endo_std	SP_raw	SP_std	Common	Genbank
1559263_s_at	30	21	28	13	383	118	FLJ46041	BG397809
229327_s_at	29	13	26	22	247	80	MAF	BE674528
207651_at	21	11	33	15	192	61	H963	NM_013308
1558740_s_at	38	20	34	17	134	7	DUSP16	R30807
217052_x_at	46	26	47	16	131	24	TIA1	AK024108
206404_at	28	16	43	15	114	26	FGF9	NM_002010
1559584_a_at	15	6	17	19	109	62	FLJ35681	BC025741
210279_at	18	11	44	42	98	17	GPR18	AF261135
219528_s_at	26	13	40	31	84	5	BCL11B	NM_022898
206761_at	25	12	26	8	82	31	CD96	NM_005816
236539_at	14	6	20	10	78	19	PTPN22	AW665758
206545_at	24	14	31	26	77	25	CD28	NM_006139
1565823_at	43	36	18	14	63	16	CDC10	BF855173
206365_at	12	6	13	4	61	5	XCL1	NM_002995
1555963_x_at	30	19	46	12	59	16	B3GNT7	CA503291
1556586_x_at	35	14	36	19	53	8	SDS3	BC041995
239157_at	23	17	42	11	52	16		AL521521
